# Mutation profiles of diffuse large B‐cell lymphoma transformation of splenic B‐cell lymphoma/leukemia, unclassifiable on whole‐exome sequencing

**DOI:** 10.1002/jha2.315

**Published:** 2021-10-13

**Authors:** Shuhei Kurosawa, Takashi Toya, Daichi Sadato, Tsunekazu Hishima, Chizuko Hirama, Yuho Najima, Takeshi Kobayashi, Kyoko Haraguchi, Yoshiki Okuyama, Keisuke Oboki, Hironori Harada, Hisashi Sakamaki, Kazuteru Ohashi, Yuka Harada, Noriko Doki

**Affiliations:** ^1^ Hematology Division Tokyo Metropolitan Cancer and Infectious Diseases Center Komagome Hospital Bunkyo City Japan; ^2^ Clinical Research Support Center Tokyo Metropolitan Cancer and Infectious Diseases Center Komagome Hospital Bunkyo City Japan; ^3^ Research Center for Genome & Medical Sciences Tokyo Metropolitan Institute of Medical Science Setagaya City Japan; ^4^ Department of Pathology Tokyo Metropolitan Cancer and Infectious Diseases Center Komagome Hospital Bunkyo City Japan; ^5^ Division of Transfusion and Cell Therapy Tokyo Metropolitan Cancer and Infectious Diseases Center Komagome Hospital Bunkyo City Japan; ^6^ Laboratory of Oncology School of Life Sciences Tokyo University of Pharmacy and Life Sciences Hachioji Japan

**Keywords:** diffuse large B‐cell lymphoma transformation, splenic B‐cell lymphoma/leukemia, unclassifiable, whole‐exome sequencing

## Abstract

A 58‐year‐old male was diagnosed with splenic B‐cell lymphoma/leukemia, unclassifiable (SPLL‐U). The lymphoma transformed into diffuse large B‐cell lymphoma (DLBCL), and multidrug chemotherapy and autologous stem cell transplantation achieved complete remission. Two years later, the lymphoma relapsed as SPLL‐U. Serial whole‐exome sequencing indicated that the mutation profiles were similar between the onset and relapsed samples while those in DLBCL were partially distinctive, which was in line with the clinical course. Hierarchical clustering revealed that an IGLL5 mutation was the founder mutation proceeding the development of the diseases and suggested that KRAS and other mutations might contribute to the transformation.

Splenic B‐cell lymphoma/leukemia, unclassifiable (SPLL‐U) is a lymphoproliferative disorder of the spleen involving small B‐cell clones which do not meet the diagnostic criteria for any other subtypes of mature B‐cell neoplasms in the WHO classification [1]. SPLL‐U includes splenic diffuse red pulp small B‐cell lymphoma (SDRPL), hairy cell leukemia‐variant (HCL‐v), and narrow sense SPLL‐U that are not classifiable as SDRPL or HCL‐v and a limited number of patients with SPLL‐U suffer from aggressive clinical courses. Recently published reports explored driver mutations in transformed B‐cell lymphoid malignancies [2, 3, 4, 5, 6]. However, to date, the data on SPLL‐U are scarce [7, 8, 9]. Herein, we reported a unique case of SPLL‐U which transformed into DLBCL. This study was performed in accordance with the Declaration of Helsinki and was approved by the Institutional Review Board of Tokyo Metropolitan Cancer and Infectious Diseases Center, Komagome Hospital, Tokyo, Japan.

A 58‐year‐old male patient presented with leukocytosis. The bone marrow (BM) was slightly hypocellular with 78.8% atypical lymphocytes. Conventional cytogenetic analyses of the BM aspirate revealed 46,XY in all metaphases. The BM biopsy revealed nodular and diffuse infiltration of lymphoid cells positive for CD20, focally and weakly positive for CD23, weakly positive for CD25, and negative for CD3, CD5, CD10, CD123, BCL‐6, Ki‐67, c‐MYC, and cyclin D1 on immunohistochemical staining (Figure 1A). These findings were not specific to any type of mature B‐cell neoplasm. The patient declined a splenectomy. The diagnosis of SPLL‐U was made (onset phase, Figure 2), and one cycle of cladribine was administered, resulting in a modest reduction of the atypical lymphocytes.

**FIGURE 1:**
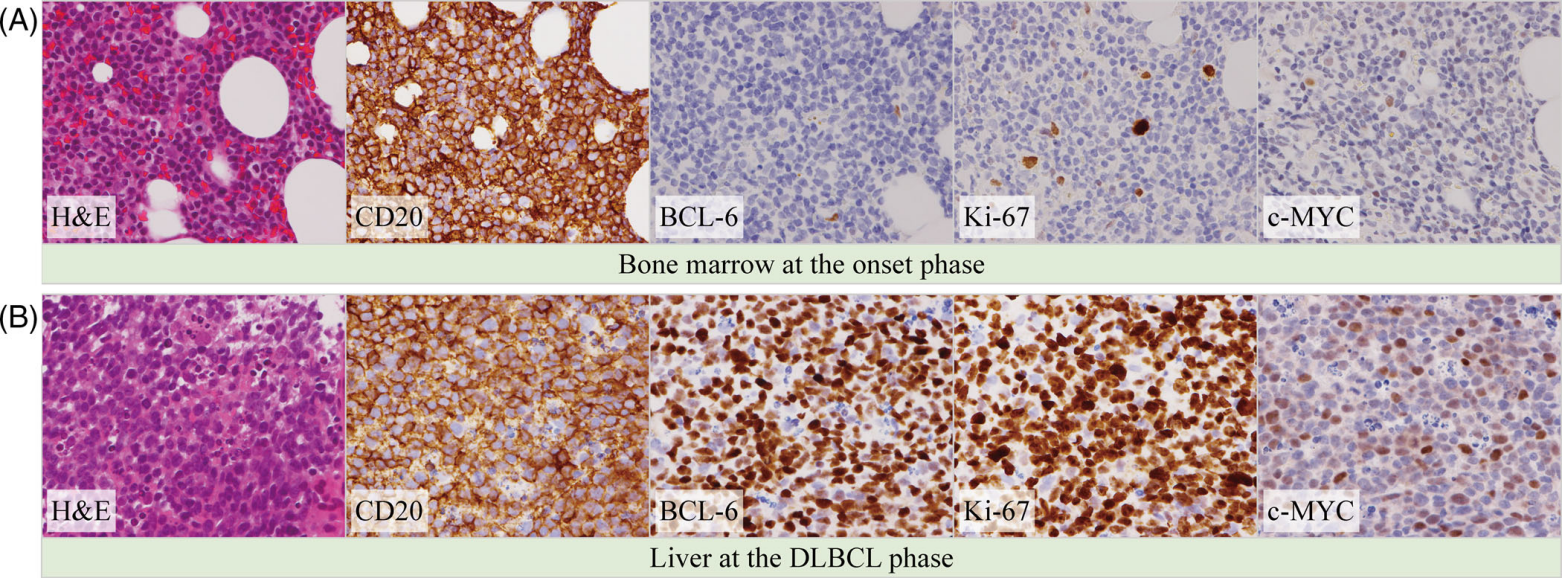
Histopathology of bone marrow at the onset phase (A) and liver at the diffuse large B‐cell lymphoma (DLBCL) phase (B). Proliferation of lymphoid cells with a nodular and diffuse pattern can be seen in the bone marrow (hematoxylin‐eosin (HE) staining). Destruction of the hepatic lobule with diffuse infiltration of lymphoid cells can be seen in the liver (HE staining). Both bone marrow (BM) and liver cells were positive for CD20 (immunohistochemistry). The BM and liver cells were negative and positive for BCL‐6, Ki‐67, and c‐MYC (immunohistochemistry), respectively

**FIGURE 2:**
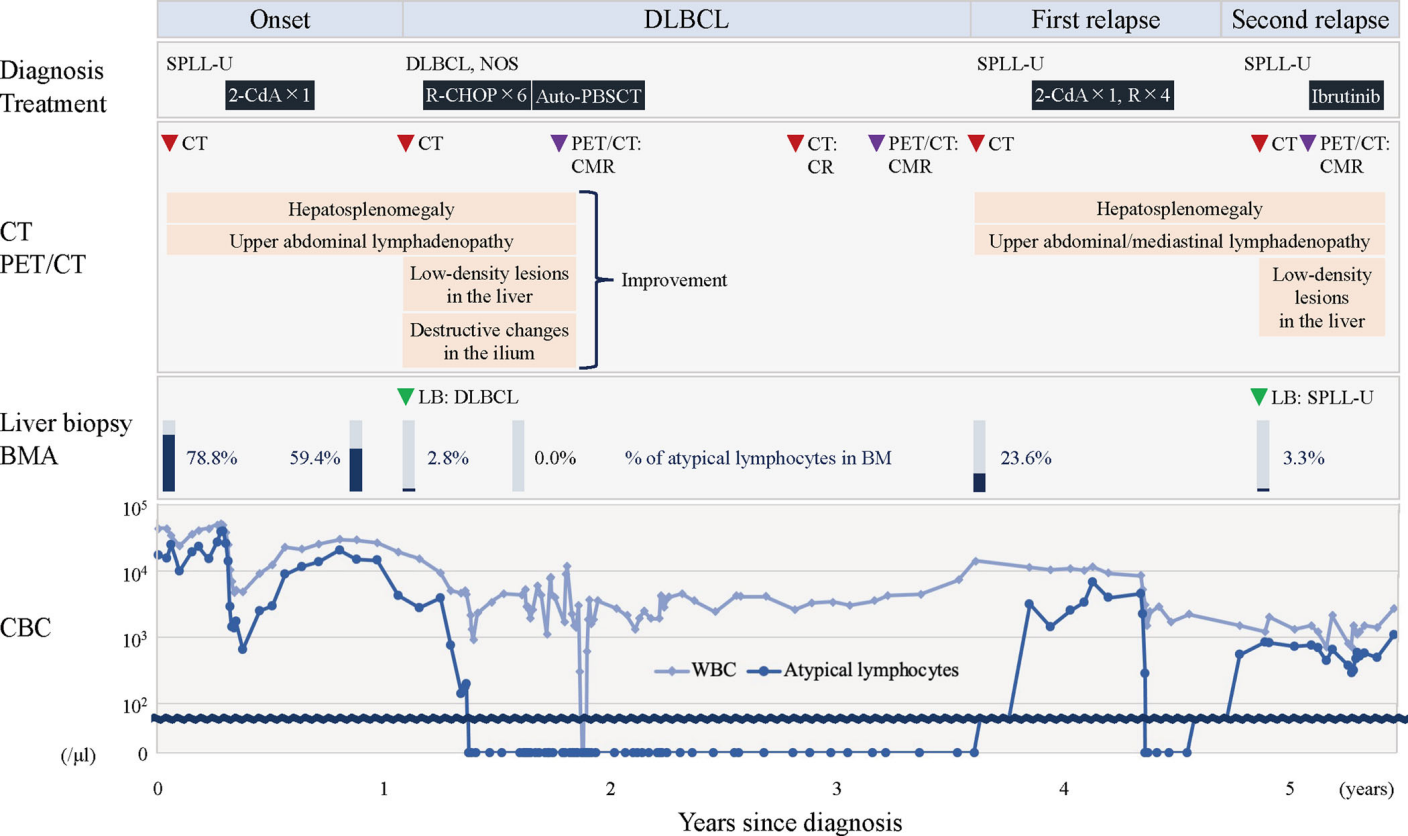
Disease time course. 2‐CdA, cladribine 0.09 mg/kg/day for 7 days; auto‐PBSCT, autologous peripheral blood stem cell transplantation; BM, bone marrow; BMA, bone marrow aspiration; CBC, complete blood count; CMR, complete metabolic response; CR, complete response; CT, computed tomography; DLBCL, diffuse large B‐cell lymphoma; Ibrutinib, ibrutinib 140 mg/day for 1 day; LB, liver biopsy; PET, positron emission tomography; R, rituximab 375 mg/m^2^ for 1 day; SPLL‐U, splenic B‐cell lymphoma/leukemia, unclassifiable; WBC, white blood cell. One course of R‐CHOP chemotherapy consisted of an intravenous cyclophosphamide 750 mg/m^2^, doxorubicin 50 mg/m^2^, vincristine 1.4 mg/ m^2^, and oral prednisolone 100 mg on days 1–5 (CHOP), with rituximab 375 mg/m^2^ infused on the day before each CHOP administration. The conditioning regimen of auto‐PBSCT consisted of rituximab 375 mg/m^2^ and melphalan 130 mg/m^2^ for 1 day, cyclophosphamide 60 mg/kg for 2 days, etoposide 500 mg/m^2^ for 3 days, and dexamethasone 39.6 mg/body for 4 days

One year later, computed tomography revealed multiple low‐density lesions throughout the liver. A liver biopsy revealed destruction of the hepatic lobule with diffuse infiltration of tumor cells positive for CD20, BCL‐6, and Ki‐67, and focally positive for c‐MYC (Figure 1B), which was consistent with DLBCL, not otherwise specified, germinal center B‐cell type (DLBCL phase, Figure 2). DLBCL cells were also found in a BM smear (0.6%) while the atypical lymphocytes had decreased (2.8%). Conventional cytogenetic analyses of the BM aspirate revealed 46,XY,add(1)(p36.1) in two of 20 metaphases. After six cycles of R‐CHOP chemotherapy and autologous peripheral blood stem cell transplantation, a complete metabolic response was confirmed on positron emission tomography/computed tomography.

Two years later, chronic lymphocytosis recurred. The flow cytometric analysis results were consistent with that in the onset phase (first relapse phase, Figure 2). Conventional cytogenetic analyses of the BM aspirate revealed 46,XY in all the metaphases. One cycle of cladribine and four cycles of rituximab were administered. Ten months later, pancytopenia and a liver nodule developed. BM aspirate revealed hypocellular marrow with CD20‐negative atypical lymphocytes, and a liver biopsy demonstrated SPLL‐U recurrence (second relapse phase, Figure 2). Ibrutinib was started, and the cytopenia gradually improved. The patient has continued receiving ibrutinib and has experienced no further recurrences.

We performed whole‐exome sequencing (WES) using DNA obtained from BM at the onset phase, the hepatic lesion at the DLBCL phase, and BM at the first and second relapse phase. Genomic DNA was extracted from each sample using the All Prep kit (Qiagen; Hilden, Germany), and WES was performed using Ion AmpliSeq Exome RDY Kit (Thermo Fisher Scientific, Waltham, MA, USA) on the Ion GeneStudio S5 system (Thermo Fisher Scientific) according to manufacturer's instructions. Sufficient amount sequence reads (mean 44 million, range: 11.9–80.9 million) were obtained and analyzed using Ion Reporter software to detect pathogenic mutations in the leukemic cells. Mean coverage depth was 135 (range: 25.86–252.6) and mean uniformity was 91% (range: 88.91–92.43) suggesting that WES was successfully worked. Detected mutations were clustered using the group average method. Curated pathogenic variants are shown in Table 1.

**TABLE 1:** Curated pathogenic variants

Time Point	Chromosome	Coordinate	Genotype	Type	Genes	Transcript	Transcript Change	Amino Acid Change	VAF	Coverage
Onset	chr12	49416372	C/A	SNV	KMT2D	NM_003482.3	c.16338+1G > T	p.?	43.23	229
Onset	chr22	23230373	G/T	SNV	IGLL5	NM_001256296.1	c.34G > T	p.Ala12Ser	40.08	237
Onset	chr3	75714935	C/A	SNV	FRG2C	NM_001124759.3	c.592C > A	p.Gln198Lys	7.54	557
Onset	chr3	75714950	C/A	SNV	FRG2C	NM_001124759.3	c.607C > A	p.Leu203Met	22.28	395
DLBCL	chr1	161047249	C/T	SNV	NECTIN4	NM_030916.2	c.724G > A	p.Val242Met	9.8	51
DLBCL	chr4	107156504	GT/G	INDEL	TBCK	NM_001163435.2	c.1370delA	p.Asn457ThrfsTer15	35	20
DLBCL	chr11	118376778	C/T	SNV	KMT2A	NM_001197104.1	c.10171C > T	p.Gln3391Ter	14.81	27
DLBCL	chr12	25380275	T/G	SNV	KRAS	NM_033360.3	c.183A > C	p.Gln61His	16.67	36
DLBCL	chr22	23230355	C/T	SNV	IGLL5	NM_001256296.1	c.16C > T	p.Gln6Ter	22.73	22
DLBCL	chr22	23230365	A/T	SNV	IGLL5	NM_001256296.1	c.26A > T	p.His9Leu	25	20
DLBCL	chr22	23230379	A/C	SNV	IGLL5	NM_001256296.1	c.40A > C	p.Thr14Pro	23.81	21
DLBCL	chr12	49431625	GC/G	INDEL	KMT2D	NM_003482.3	c.9513delG	p.Pro3172HisfsTer25	29.03	31
DLBCL	chr3	48680471	G/A	SNV	CELSR3	NM_001407.2	c.8335C > T	p.Arg2779Trp	7.55	53
DLBCL	chr4	106157167	C/T	SNV	TET2	NM_001127208.2	c.2068C > T	p.Gln690Ter	7.41	54
DLBCL	chr4	185018473	C/T	SNV	ENPP6	NM_153343.3	c.1042G > A	p.Gly348Ser	8.51	47
DLBCL	chr5	40692160	C/T	SNV	PTGER4	NM_000958.2	c.1147C > T	p.Arg383Trp	7.84	51
DLBCL	chr6	54805306	G/A	SNV	FAM83B	NM_001010872.2	c.1537G > A	p.Gly513Arg	13.95	43
DLBCL	chr7	150846025	G/A	SNV	GBX1	NM_001098834.2	c.743C > T	p.Ala248Val	11.43	35
DLBCL	chr10	5929864	G/A	SNV	ANKRD16	NM_019046.2	c.481C > T	p.Pro161Ser	6.94	72
DLBCL	chr12	25380275	T/G	SNV	KRAS	NM_033360.3	c.183A > C	p.Gln61His	16.67	36
DLBCL	chr12	70824288	G/A	SNV	KCNMB4	NM_014505.5	c.488G > A	p.Arg163His	13.89	36
DLBCL	chr15	42985912	G/A	SNV	STARD9	NM_020759.2	c.12136G > A	p.Gly4046Ser	17.14	35
DLBCL	chr17	7579340	G/A	SNV	TP53	NM_000546.5	c.347C > T	p.Ser116Phe	11.11	36
DLBCL	chr17	20916179	C/T	SNV	USP22	NM_015276.1	c.908G > A	p.Gly303Asp	9.43	53
DLBCL	chr19	38828038	G/T	SNV	CATSPERG	NM_021185.4	c.164G > T	p.Arg55Met	18.18	22
DLBCL	chr20	56188345	G/A	SNV	ZBP1	NM_030776.2	c.544C > T	p.Gln182Ter	8	50
DLBCL	chr22	23230373	G/T	SNV	IGLL5	NM_001256296.1	c.34G > T	p.Ala12Ser	15	20
First relapse	chr12	49416372	C/A	SNV	KMT2D	NM_003482.3	c.16338+1G > T	p.?	34.01	441
First relapse	chr22	23230373	G/T	SNV	IGLL5	NM_001256296.1	c.34G > T	p.Ala12Ser	43.1	536
First relapse	chr3	75714950	C/A	SNV	FRG2C	NM_001124759.3	c.607C > A	p.Leu203Met	16.55	840
First relapse	chr3	75714950	C/A	SNV	FRG2C	NM_001124759.3	c.607C > A	p.Leu203Met	16.55	840
First relapse	chr12	122359408	G/A	SNV	WDR66	NM_144668.5	c.197G > A	p.Gly66Glu	11.76	51
First relapse	chr21	14982716	T/C	SNV	POTED	NM_174981.3	c.167T > C	p.Met56Thr	6.48	108
Second relapse	chr12	49416372	C/A	SNV	KMT2D	NM_003482.3	c.16338+1G > T	p.?	15.63	435
Second relapse	chr22	23230373	G/T	SNV	IGLL5	NM_001256296.1	c.34G > T	p.Ala12Ser	18.76	453
Second relapse	chr3	75714935	C/A	SNV	FRG2C	NM_001124759.3	c.592C > A	p.Gln198Lys	5.68	1092
Second relapse	chr3	75714950	C/A	SNV	FRG2C	NM_001124759.3	c.607C > A	p.Leu203Met	13.41	753
Second relapse	chr12	49416372	C/A	SNV	KMT2D	NM_003482.3	c.16338+1G > T	p.?	12.87	272

Abbreviation: DLBCL, diffuse large B‐cell lymphoma.

Detected variants were annotated with transcript and protein location information according to HGVS‐nomenclature. And more, information from the 1000G, Exac, cosmic, and CLINVAR databases was also added. To exclude SNPs, variants with a prevalence greater than 1% in a given regional population (using 1000G and Exac) were excluded. Variants which previously reported as myeloid‐ or lymphoid‐ associated mutations given by cosmic and CLINVAR were further selected as candidate pathogenic mutations. All annotation was performed using Ion Reporter software (Thermo Fisher Scientific) which contains the above databases. Finally, candidate mutations were manually curated by molecular hematologists.

In line with the clinical findings, the mutation patterns were generally similar between the onset relapse phase while the mutation profiles in the DLBCL phase partially differed from the others (Figures 3A,B). In the hierarchical clustering, an *IGLL5* mutation was detected in all the samples, suggesting that these founder mutations preceded the development of the disease. *IGLL5* mutations, which are critical for B‐cell development, were frequently detected in various B‐cell lymphoid malignancies [10, 11]. These mutations were reported to be linked to canonical activation induced‐cytidine deaminase (AID) activity [11]. Although AID normally contributes to the diversity of antibodies by introducing somatic mutations in immunoglobulin genes, off‐target AID activity could induce genomic instability and initiate oncogenesis [12]. The *IGLL5* mutations might have played a key role also in the present case.

**FIGURE 3:**
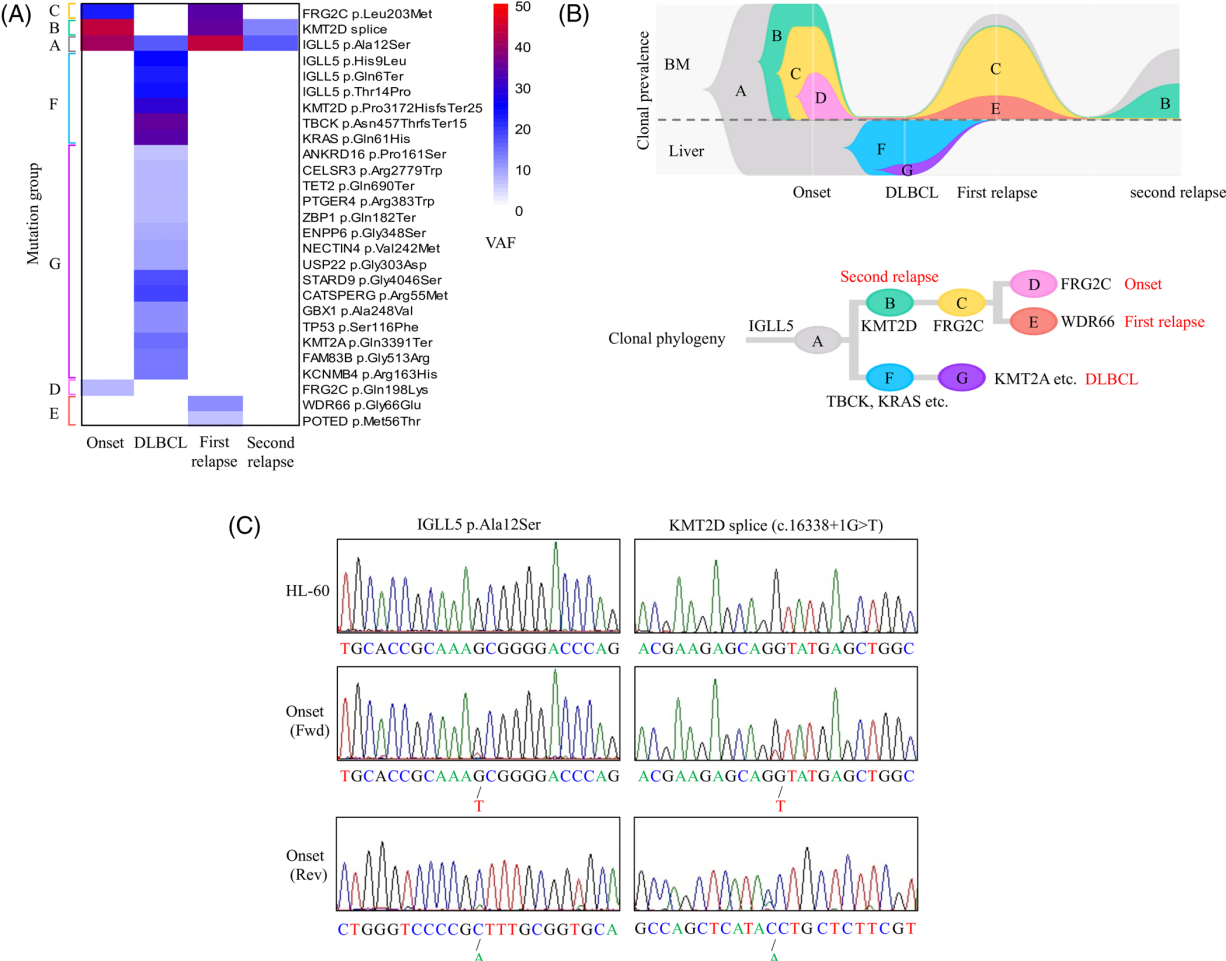
(A) Hierarchical clustering by whole‐exome sequencing. Mutation profiles in each phase were described. The color indicates the variant allele frequency of each mutation as shown in the figure. Mutations were classified into seven groups according to hierarchical cluster analysis. Names of the clusters correspond to the clones in (B). (B) Fish plot showing the clonal transition of tumor cells. BM, bone marrow; DLBCL, diffuse large B‐cell lymphoma. (C) Sanger sequencing results of the *IGLL5* and *KMT2D* mutations in the onset sample. Results from HL‐60 were used as the non‐mutated control. The result of the onset sample was shown in both forward (Onset Fwd) and reverse sequence (Onset Rev)

Additionally, several types of *KMT2D* mutations were found in all the samples. KMT2D is a histone H3 lysine 4 methyltransferase promoting chromatin opening and transcriptional activation of the targeted genes [13]. It frequently shows mutations in various lymphoid malignancies [10, 13–16]. To our knowledge, the present study is the first to detect *KMT2D* mutations in SPLL‐U although the exact reason why different *KMT2D* mutations appeared alternately was unclear. Further investigation is warranted to explore the underlying pathogenesis in SPLL‐U. Sanger sequencing confirmed the presence of IGLL5 p.Ala12Ser and KMT2D c.16338+1G > T mutations with a lower allelic ratio than that detected from WES, possibly result by PCR bias (Figure 3C).

Several studies explored driver mutations in transformed aggressive B‐cell lymphoid malignancies by next‐generation sequencing (Table 2) [2, 3, 4, 5, 6]. In the present case, *KRAS* mutation, which drives aggressive cell proliferation, was found at the DLBCL phase and might have contributed to the progression of SPLL‐U into DLBCL in a process similar to that reported in Richter syndrome [6].

**TABLE 2:** Mutations in transformed aggressive B‐cell lymphoid malignancies from literature review

Author, year	Primary disease	No. of serial samples	NGS	Mutations detected in transformed cases
Fabbri et al., 2013	CLL	9	WES	*TP53* and *NOTCH1*
Kiel et al., 2012	SMZL	6	WGS	*NOTCH2*
Bouska et al., 2017	FL	12	WES	*MYC*, *EBF1*, *IRF4*, *RPN1*, *SOCS1*, *SYNE1*, *SGK1*, *PIM1*, *EP300*, *BMP7*, *ETS1*, *SARDH*, *TAF1*, *FBX011* and *HIST1H1E* etc.
Vogelsberg et al., 2020	ISFN	10	Targeted sequencing	*TP53*, *CD79B*, and *HIST1H1B* etc.
Klintman et al., 2021	CLL	17	WGS	*TP53*, *XPO1*, *NOTCH1*, *SF3B1*, *BIRC3*, *ATM*, *RPS15*, *BRAF*, *KRAS*, *TRAF3*, *SETD2*, *PTPN11*, *MGA*, and *BAZ2A* etc.

Abbreviations: CLL, chronic lymphocytic leukemia; FL, follicular lymphoma; ISFN, in situ follicular neoplasia; NGS, next‐generation sequencing; SMZL, splenic marginal zone lymphoma; WES, whole‐exome sequencing; WGS, whole‐genome sequencing.

Our study has some limitations: it was a single case report, and the unavailability of spleen samples analysis prevented the identification of specific SPLL‐U types (SDRPL, HCL‐v, and narrow sense SPLL‐U) and SMZL [7]. However, the WHO classification defines SPLL‐U as a provisional entity requiring additional molecular studies [1]. The present case provides an important insight into the pathogenesis of SPLL‐U despite the limited evidence available. We also note that the percentage of atypical lymphocytes in the microscopic examination could be lower than VAFs in WES because not only apparently malignant cells harbor mutations, as previously reported [17].

In summary, we described the clinical presentation, immunophenotype, and genetic landscape of a case of DLBCL transformation of SPLL‐U. Serial analyses using next‐generation sequencing have the potential to provide useful information about the origins and progression of this uncommon disease. More patient data and prospective studies are needed to deepen our understanding of SPLL‐U pathophysiology.

## CONFLICTS OF INTEREST

The authors declare that they have no conflict of interest.

## References

[1] Piris MA , Campo E , Foucar K , Falini B , Mollejo M , Swerdlow H , et al . Splenic B‐cell lymphoma/leukaemia, unclassifiable. , In: WHO classification of tumours of haematopoietic and lymphoid tissues. Revised 4th ed. IARC: Lyon; 2017. p. 229–31.

[2] Fabbri G , Khiabanian H , Holmes AB , Wang J , Messina M , Mullighan CG , et al. Genetic lesions associated with chronic lymphocytic leukemia transformation to Richter syndrome. J Exp Med. 2013;210(11):2273–88. 24127483 10.1084/jem.20131448 PMC3804949

[3] Kiel MJ , Velusamy T , Betz BL , Zhao L , Weigelin HG , Chiang MY , et al. Whole‐genome sequencing identifies recurrent somatic NOTCH2 mutations in splenic marginal zone lymphoma. J Exp Med. 2012;209(9):1553–65. 22891276 10.1084/jem.20120910 PMC3428949

[4] Bouska A , Zhang W , Gong Q , Iqbal J , Scuto A , Vose J , et al. Combined copy number and mutation analysis identifies oncogenic pathways associated with transformation of follicular lymphoma. Leukemia 2017;31(1):83–91. 27389057 10.1038/leu.2016.175 PMC5214175

[5] Vogelsberg A , Steinhilber J , Mankel B , Federmann B , Schmidt J , Montes‐Mojarro IA , et al. Genetic evolution of in situ follicular neoplasia to aggressive B‐cell lymphoma of germinal center subtype. Haematologica 2020. In press:10.3324/haematol.2020.254854. 10.3324/haematol.2020.254854 PMC8485666 32855278

[6] Klintman J , Appleby N , Stamatopoulos B , Ridout K , Eyre TA , Robbe P , et al. Genomic and transcriptomic correlates of Richter transformation in chronic lymphocytic leukemia. Blood 2021;137(20):2800–16. 33206936 10.1182/blood.2020005650 PMC8163497

[7] Suzuki T , Miyoshi H , Shimono J , Kawamoto K , Arakawa F , Furuta T , et al. Clinicopathological analysis of splenic red pulp low‐grade B‐cell lymphoma. Pathol Int. 2020;70(5):280–6. 32052529 10.1111/pin.12909

[8] Matutes E , Martinez‐Trillos A , Campo E . Hairy cell leukaemia‐variant: disease features and treatment. Best Pract Res Clin Haematol. 2015;28(4):253–63. 26614904 10.1016/j.beha.2015.09.002

[9] Zanelli M , Ragazzi M , Valli R , Piattoni S , De Celis MIA , Farnetti E , et al. Transformation of IGHV4‐34+ hairy cell leukaemia‐variant with U2AF1 mutation into a clonally‐related high grade B‐cell lymphoma responding to immunochemotherapy. Br J Haematol. 2016;173(3):491–5. 26303517 10.1111/bjh.13627

[10] Jaramillo Oquendo C , Parker H , Oscier D , Ennis S , Gibson J , Strefford JC . Systematic review of somatic mutations in splenic marginal zone lymphoma. Sci Rep. 2019;9(1):10444. 31320741 10.1038/s41598-019-46906-1 PMC6639539

[11] Kasar S , Kim J , Improgo R , Tiao G , Polak P , Haradhvala N , et al. Whole‐genome sequencing reveals activation‐induced cytidine deaminase signatures during indolent chronic lymphocytic leukaemia evolution. Nat Commun. 2015;6:8866. 26638776 10.1038/ncomms9866 PMC4686820

[12] Keim C , Kazadi D , Rothschild G , Basu U . Regulation of AID, the B‐cell genome mutator. Genes Dev. 2013;27(1):1–17. 23307864 10.1101/gad.200014.112 PMC3553278

[13] Zhang Q , Wang HY , Liu X , Roth MH , Shestov AA , Lee S‐C , et al. Dynamic changes in gene mutational landscape with preservation of core mutations in mantle cell lymphoma cells. Front Oncol. 2019;9:568. 31334109 10.3389/fonc.2019.00568 PMC6617136

[14] Okosun J , Bodor C , Wang J , Araf S , Yang C‐Y , Pan C , et al. Integrated genomic analysis identifies recurrent mutations and evolution patterns driving the initiation and progression of follicular lymphoma. Nat Genet. 2014;46(2):176–81. 24362818 10.1038/ng.2856 PMC3907271

[15] Ortega‐Molina A , Boss IW , Canela A , Pan H , Jiang Y , Zhao C , et al. The histone lysine methyltransferase KMT2D sustains a gene expression program that represses B cell lymphoma development. Nat Med. 2015;21(10):1199–1208. 26366710 10.1038/nm.3943 PMC4676270

[16] Li Q , Zhang W , Li J , Xiong J , Liu J , Chen T , et al. Plasma circulating tumor DNA assessment reveals *KMT2D* as a potential poor prognostic factor in extranodal NK/T‐cell lymphoma. Biomark Res. 2020;8:27. 32695399 10.1186/s40364-020-00205-4 PMC7366898

[17] Marsilio S , Khiabanian H , Fabbri G , Vergani S , Scuoppo C , Montserrat E , et al. Somatic CLL mutations occur at multiple distinct hematopoietic maturation stages: documentation and cautionary note regarding cell fraction purity. Leukemia 2018;32(4):1041–4. 29203856 10.1038/leu.2017.343 PMC5886053

